# Binding of *Helicobacter pylori* to Human Gastric Mucins Correlates with Binding of TFF1

**DOI:** 10.3390/microorganisms6020044

**Published:** 2018-05-18

**Authors:** Ciara Dunne, Julie Naughton, Gina Duggan, Catherine Loughrey, Michelle Kilcoyne, Lokesh Joshi, Stephen Carrington, Helen Earley, Steffen Backert, Catherine Robbe Masselot, Felicity E. B. May, Marguerite Clyne

**Affiliations:** 1 School of Medicine and Conway Institute, University College Dublin, Belfield, Dublin 4, D04 V1W8, Ireland; ciaradunne51@gmail.com (C.D.); g.duggan@imperial.ac.uk (G.D.); helen.earley@ucdconnect.ie (H.E.); 2 School of Biological Sciences, Dublin Institute of Technology, Kevin St., Dublin 2, D08 NF82, Ireland; julieann.naughton@dit.ie; 3 Glycoscience Group, National Centre for Biomedical Engineering Science, National University of Ireland Galway, Galway, H91 TK33, Ireland; catherine.loughrey@nuigalway.ie (C.L.); Michelle.kilcoyne@nuigalway.ie (M.K.); lokesh.joshi@nuigalway.ie (L.J.); 4 Carbohydrate Signalling Group, Microbiology, School of Natural Sciences, National University of Ireland Galway, Galway, H91 TK33, Ireland; 5 Veterinary Science Centre, University College Dublin, Belfield, Dublin 4, D04 V1W8, Ireland; Stephen.carrington@ucd.ie; 6 Division of Microbiology, Department of Biology, Friedrich Alexander University Erlangen-Nuremberg, Staudtstr. 5, D-91058 Erlangen, Germany; steffen.backert@fau.de; 7 Unité de Glycobiologie Structurale et Fonctionnelle, University Lille Nord de France, USTL, UGSF, IFR 147, CNRS, UMR 8576, F-59650 Villeneuve d’Ascq, France; catherine.robbe@univ-lille1.fr; 8 Northern Institute for Cancer Research, University of Newcastle Upon Tyne, Newcastle Upon Tyne NE2 4HH, UK; F.E.B.May@ncl.ac.uk

**Keywords:** *Helicobacter pylori*, TFF1, mucin, lectin

## Abstract

*Helicobacter pylori* binds to the gastric mucin, MUC5AC, and to trefoil factor, TFF1, which has been shown to interact with gastric mucin. We examined the interactions of TFF1 and *H. pylori* with purified gastrointestinal mucins from different animal species and from humans printed on a microarray platform to investigate whether TFF1 may play a role in locating *H. pylori* in gastric mucus. TFF1 bound almost exclusively to human gastric mucins and did not interact with human colonic mucins. There was a strong correlation between binding of TFF1 and *H. pylori* to human gastric mucins, and between binding of both TFF1 and *H. pylori* to gastric mucins with that of *Griffonia simplicifolia* lectin-II, which is specific for terminal non-reducing α- or β-linked *N*-acetyl-d-glucosamine. These results suggest that TFF1 may help to locate *H. pylori* in a discrete layer of gastric mucus and hence restrain their interactions with epithelial cells.

## 1. Introduction

*Helicobacter pylori* is one of the most common infections of mankind, as half of the human world population carries the bacterium. It has evolved with humans over thousands of years [1] and is uniquely adapted to colonize the hostile environment of the stomach. Infection with *H. pylori* usually occurs early in childhood and lasts for the lifetime of the host unless eradicated with antimicrobials [2]. In some patients, infection can cause the development of gastritis and duodenal ulceration [3] and chronic infection is also associated with an increased risk of developing gastric cancer in later life [4]. *H. pylori* has been classified as a class I carcinogen [5]. One of the striking characteristics of *H. pylori* is that it displays a strict host and tissue specificity. Natural infection with *H. pylori* only occurs in humans and other primates. In addition, the only well-established reservoir for *H. pylori* is the stomach where the majority of the colonizing organisms are found in the gastric mucus layer overlying the epithelial cells [6]. The organisms display a strong tropism for the gastric mucin MUC5AC, which they bind to via *O*-linked oligosaccharides expressed on the variable number tandem repeat (VNTR) side chain of the mucin. Specific carbohydrate ligands bound by *H. pylori* include the Lewis^b^ (Le^b^) blood group antigen [7,8], the H-1 type blood group antigen [7], *N*,*N*′- diacetyllactosediamine (LacdiNAc) [9], and sialylated structures such as sialyl-Le^x^ and sialyl-Le^a^ [10]. Strains of *H. pylori* that express *H. pylori* outer membrane proteins, the BabA and/or SabA adhesins, bind to Le^b^ [11] and to sialylated structures [10], respectively. The outer membrane protein adhesin LabA has been shown to bind to LacdiNAc, a structure, which is also expressed on MUC5AC [9]. Binding of *H. pylori* to gastric mucins therefore is determined both by the mucin glycosylation and also by the adhesins expressed by individual strains.

We have previously shown that *H. pylori* can interact with TFF1 [12,13], one of three members of the trefoil factor family of proteins found in humans. TFF1 is found in gastric mucus and is co-expressed with MUC5AC in foveolar pit cells of the gastric body and superficial regions of the antral glands [14]. In the human gastric cytosol, TFF1 is found in three molecular forms: as TFF1 monomer, TFF1 homodimer and as a 25 kDa heterodimer in which the TFF1 monomer is bound covalently to an 18 kDa BRICHOS domain-containing protein, gastrokine 2 [15,16]. The TFF1 dimer comprises two TFF1 monomer units each folded in the characteristic trefoil motif [15,16] and connected by a flexible linker consisting of the two C-terminal regions with an intermolecular disulfide bond between the Cys^58^ residues [17]. Two putative receptor ligand recognition domains of the TFF1 homodimer are located at opposite ends of the flexible linker [18]. The variable distance and orientation of the TFF1 binding sites offer versatility and suggests that TFF1 may bind to receptor binding sites on two different proteins. TFF1 is intimately associated with gastric mucus [15], and in vivo, the majority of TFF1 dimer and some TFF1 monomer bind firmly but non-covalently to soluble gastric mucins [19]. The core-oligosaccharide portion of rough-form (RF) lipopolysaccharide (LPS) mediates binding of *H. pylori* to TFF1 [13,20]. TFF1 binds to bovine serum albumin- (BSA-)conjugated mannose and glucose, and free mannose and glucose inhibits binding of *H. pylori* LPS to TFF1 [13] which indicates the carbohydrate mediated nature of TFF1 binding. Optimal binding of *H. pylori* LPS to TFF1 occurs at pH 5.0 to 6.0 [13], which could promote colonization of the mucus layer adjacent to the gastric epithelial surface, away from the lumen where the bacteria are likely to be removed with mucus flow. A mutant of *H. pylori* that expresses a truncated LPS is unable to bind TFF1, and compared to the parental wild type strain, has the reduced ability to colonize an adherent mucus layer that contains TFF1 and MUC5AC [20].

Given the strict host specificity of *H. pylori* and its tropism for the gastric mucin, MUC5AC, and in light of our previous finding that *H. pylori* binds to TFF1 [13], we examined the interaction of TFF1 with human and animal mucins to investigate whether TFF1 might play a role in facilitating *H. pylori* colonization of gastric mucus. Our results show that TFF1 binds exclusively to gastric mucins and there is a direct correlation between binding of *H. pylori* and binding of TFF1 to gastric mucins. These results suggest that TFF1 may play a role in retaining the majority of *H. pylori* organisms in a specific stratum of gastric mucus rather than at the epithelial cell surface, which would limit inflammation and promote chronic infection. 

## 2. Materials and Methods 

### 2.1. Bacterial Strains and Culture Conditions

*H. pylori* strains P12 [21], 26695 [22], J99 [23] and G27 [24] were used in this study. *Campylobacter jejuni* strains used were the invasive strain, 81-176 [25,26] and the less invasive strain, NCTC11168 [27]. *H. pylori* strains were cultured on Columbia blood agar base containing 7% (*v*/*v*) horse defibrinated blood. *C. jejuni* strains were cultured at 37 °C on Mueller-Hinton (MH) agar. Microaerophilic conditions were generated using CampyGen gas packs (Oxoid).

### 2.2. Harvest and Purification of Animal and Human Gastric and Colonic Mucins

Animal mucins were collected and purified as described previously [28,29]. Human mucins were isolated from gastric and colonic tissue. Mucins were purified from healthy gastric tissue of twelve different individuals as described previously [30] and were labelled GM1–GM12. The blood group status of individuals from whom gastric tissue was taken from is listed in Supplementary Table S1. Colonic biopsies (approximately 2 cm^2^) resected from excised colon and colonic mucins, labeled CM1–CM9, were isolated from healthy tissue of individuals with colorectal cancer, while ulcerative colitis mucins, labeled UCM1–UCM5, were isolated from inflamed tissue of individuals with acute ulcerative colitis who were undergoing colectomies as previously described [31]. Ethical approval was obtained from the Ethics and Medical Research Committee in St. Vincent’s University Hospital, Dublin (AMD 8 JUN 12, 08/06/2012). Prior to the procedure and sample collection, all patients gave informed, written consent, and all experimental methods involving human tissues were performed in accordance with the ethics committee guidelines and regulations. Briefly, mucus samples from either animal or human tissues were collected by scraping of mucosal surfaces with a scalpel blade to harvest secreted mucus and epithelial cells. Mucus was solubilized with guanidine hydrochloride (final concentration, 4 M). Samples were reduced with dithiothreitol (DTT) (Sigma-Aldrich Co., Dublin, Ireland) at a final concentration of 0.01 M for 5 h at 37 °C and were alkylated with iodoacetamide (0.025 M) (Sigma-Aldrich). Mucin was purified using CsCl density gradient separation followed by size exclusion chromatography. Mucin-rich fractions identified by slot blotting and staining with periodic acid-Schiffs (PAS) reagent were pooled.

### 2.3. Construction of Animal and Human Gastrointestinal Tract (GIT) Mucin Microarrays

Microarrays containing 38 purified mucins from the GITs of eight different animal species and two mucins from colonic cell lines, E12 [32] and LS174T [33], (Supplementary Table S2) were constructed as previously described [28]. Purified human gastric and colonic mucins (Supplementary Table S3) were printed onto separate microarray slides as described previously [28]. Briefly, the mucins were dissolved in phosphate buffered saline, pH 7.4, (PBS) to a final concentration of 0.5 mg/mL in PBS with or without a range of concentrations of detergent (Supplementary Tables S2 and S3). The mucins were printed onto Nexterion^®^ slide H microarray slides (Schott AG, Mainz, Germany) using a SciFLEXARRAYER S3 (Scienion AG, Berlin, Germany). Each mucin was printed in replicates of six per subarray, with eight identical subarrays per slide. Slides were incubated in a humid atmosphere overnight and active functional groups were deactivated by incubated in 100 mM ethanolamine in 50 mM sodium borate, pH 8.0, for 1 hr. Slides were then washed three times in PBS supplemented with 0.05% Tween 20 (PBST), once in PBS, centrifuged dry (1500 rpm, 5 min) and stored with desiccant at 4 °C until use.

### 2.4. Profiling of Mucin Microarrays with Lectins 

To analyze the glycosylation profiles of the immobilized human mucins, the microarray slides were incubated with a panel of tetramethylrhodamine isothiocyanate (TRITC)-labeled lectins (Supplementary Table S4). All incubation procedures were carried out in the dark. Lectins were incubated at the indicated concentration in TBST (Supplementary Table S4) on the mucin using an eight well gasket and incubation cassette system (Agilent Technologies, Dublin, Ireland) for 1 h at 37 °C in the dark with gentle rotation (4 rpm). Following incubation, slides were washed three times in low salt Tris buffered saline (20 mM Tris, 100 mM NaCl, 1 mM CaCl_2_, 1 mM MgCl_2_, pH 7.2, TBS) supplemented with 0.05% Tween 20 (TBST), then once in TBS and once in deionized water and dried by centrifugation as above. Microarrays were scanned immediately in an Agilent G2505B microarray scanner (532 nm laser, 90% PMT and 5 μm resolution). Images were saved as *.tif files for data extraction.

### 2.5. Profiling of Mucin Microarrays with TFF1

Recombinant human TFF1 dimer [17] at 1, 5, 10 and 50 µg/mL in PBS were incubated on the mucin microarrays for 1 h at 37 °C with gentle rotation (4 rpm). Slides were washed three times in TBST, once in TBS and then centrifuged dry. Dried slides were incubated with a monoclonal TFF1 antibody, 14H3, in a 1 in 100 dilution in TBS for 1 h at 37 °C. Slides were washed and dried as previously described and then incubated with a Cy5-labelled goat anti-mouse antibody for 1 h at 37 °C in the dark at 4 rpm. Slides were then washed three times in TBST, and once in TBS [28]. The microarrays were dried by centrifugation and scanned immediately in a GenePix 4000b microarray scanner (Molecular Devices, San Jose, CA, USA) using the 532 nm laser (100% laser power, 70% photomultiplier tube (PMT), 5 µm resolution) and also the 635 nm laser (100% laser power, 70% PMT, 5 µm resolution). TFF1 incubations were conducted in duplicate per microarray slide. For quality control, two subarrays on each slide were incubated separately with TRITC-labeled lectins *Artocarpus integrifolia* agglutinin (AIA) and *Ulex europaeus* agglutinin I (UEA-I) to ensure the integrity and stability of the immobilized mucins.

### 2.6. Interrogation of Human Mucin Microarrays with H. pylori and with C. jejuni

*C. jejuni* and *H. pylori* cultured on agar as described above were harvested, and resuspended in MH broth or Brain Heart Infusion (BHI) broth supplemented with 10% fetal bovine serum (FBS), respectively, to an OD_600nm_ of 0.2. *C. jejuni* cultures were incubated for 4 h, and *H. pylori* cultures for 16 h at 37 °C and 200 rpm at which time bacteria were harvested, washed twice in PBS, and resuspended to an OD_600nm_ of 1.0 in PBS. Bacteria were labelled with the fluorescent dye SYTO 82^®^ as previously described and resuspended in TBST [29]. Microarrays were incubated with fluorescently labelled bacteria diluted in TBST as described above. *H. pylori* incubations were conducted in duplicate per microarray slide. All experiments were performed on three separate occasions. Two subarrays per experiment were incubated with TRITC-labelled AIA and UEA-I for quality control. After incubations, slides were washed and dried as described above, and scanned immediately using the 532 nm laser in a GenePix 4000b microarray scanner (Molecular Devices). 

### 2.7. Microarray Data Extraction and Analysis

Fluorescence intensity data was extracted from .tif files using GenePix Pro v.5.1 software (Molecular Devices) and a proprietary *.gal file using adaptive diameter (70–130%) circular alignment based on 230 μm features and exported to Excel (v.2010, Microsoft, Redmond, WA, USA) as a text file, where subsequent data processing and analysis was performed. Local background was subtracted, and background corrected median feature intensity (F532median-B532 or F635median-B635) was used for each feature intensity value. The median of six replicate features per subarray was handled as a single data point for graphical and statistical analysis. Data were normalized to the mean for three replicate microarray slides subarray by subarray using subarray total intensity mean. Binding data was presented as a bar graph of mean intensity with an error bar of one standard deviation of three experimental replicates or in cases of one experiment as a bar graph of median intensity of the six technical replicates.

### 2.8. Statistical and Hierarchical Clustering Analysis

The Mann-Whitney U test was used to estimate statistical significance between two different conditions, with a *p*-value of <0.05 considered significant. Bar charts of *H. pylori* and TFF1 binding of human mucins were generated using GraphPad Prism, version 5 (La Jolla, CA, USA). To determine the correlation between *H. pylori*, TFF1, and lectin binding of human gastric and colonic mucins, a log transformation of each data set was performed prior to plotting and calculation of the Spearman correlation coefficient using the R statistical software environment, version 1.1.442, (Vienna, Austria). 

Lectin data scaled to a fluorescence intensity maximum of 50,000 relative fluorescence units (RFU) was subjected to unsupervised clustering with complete linkage and Euclidean distance using Hierarchical Clustering Explorer v3.0 (HCE 3.0, University of Maryland [34]).

## 3. Results

### 3.1. TFF1 Interacts Specifically with Human and Animal Gastric Mucins

**Figure 1:**
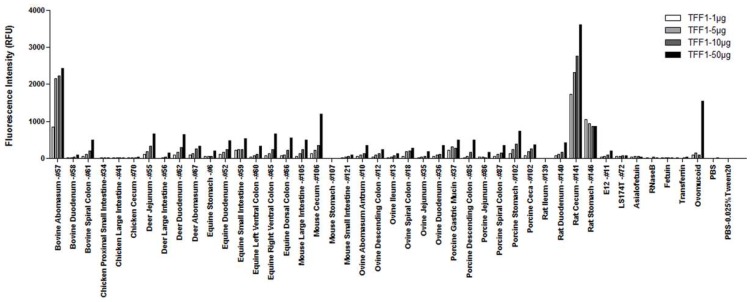
TFF1 binding to purified animal mucins. Mucin microarrays containing gastrointestinal mucins from eight different animal species were incubated with different concentrations of TFF1 dimer (1, 5, 10, and 50 µg/mL). Results presented are from one experiment, with the binding intensity indicated by the relative fluorescence intensity (median result from six technical replicates).

We have previously shown that both *H. pylori* and *C. jejuni*, two closely related species, have distinct binding profiles when tested against a panel of animal mucins printed on a microarray [29]. We therefore used mucin microarrays containing 38 mucins from eight different animal species and two mucins from colonic cell lines, E12 and LS174T, to do a preliminary assessment of TFF1 binding (Figure 1). The microarrays were probed with varying concentrations of recombinant human TFF1 dimer. TFF1 bound with highest intensity to animal mucins isolated from bovine abomasum, rat cecum and rat stomach. These were the same rat and bovine mucins that we previously demonstrated to show *H. pylori* binding [29]. Binding was TFF1 concentration dependent for all binding except for the rat stomach mucin. Lower, but concentration-dependent, binding was also observed for mucins from bovine spiral colon, deer jejunum, deer duodenum, deer abomasum, equine duodenum, equine small intestine, equine left and right ventral colons, equine dorsal colon, mouse large intestine, mouse cecum, ovine abomasum antrum, ovine descending colon, ovine spiral colon, ovine duodenum, porcine gastric mucin, porcine descending colon, porcine spiral colon, porcine stomach, porcine ceca, and rat duodenum. TFF1 did not interact with mucin from either the E12 or LS174T cell lines. Interestingly, TFF1 bound to ovomucoid from chicken egg only at the highest concentration incubated, 50 μg/mL (Figure 1). 

**Figure 2:**
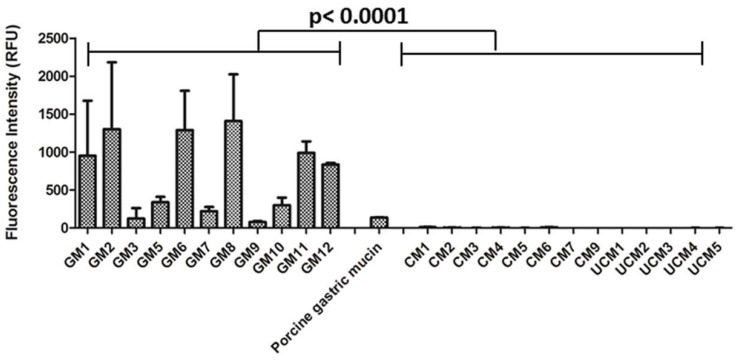
Binding of TFF1 dimer to gastric and colonic mucins. Mucin microarrays containing human gastric (GM) and colonic mucins (CM) were incubated with TFF1 dimer (20 µg/mL). GM = gastric mucin, CM = colonic mucin, UCM = ulcerative colitis mucin. GM4 was not printed on these microarrays. Results are presented as the mean of three experimental replicates ± one standard deviation, with the binding intensity indicated by the relative fluorescence intensity. The level of TFF1 binding to gastric mucins was significantly higher than TFF1 binding to colonic mucins (*p* < 0.0001, Mann-Whitney U test).

Human gastric and colonic mucin microarrays demonstrated that TFF1 bound exclusively to human gastric mucins (Figure 2). Considerable variation in TFF1 binding to different gastric mucins was observed. The highest binding occurred with gastric mucin 8 (GM8) and strong binding to GM1, GM2, GM6, GM11 and GM12 was also noted. The donors of GM1 and GM2 were secretors and produced the Le^b^ blood group antigen, and donors of GM1, GM6, GM8, GM11 and GM12 were blood group O (Table S1). Weak binding of TFF1 to porcine gastric mucin was also detected consistent with that observed on the animal mucin microarray. Remarkably, TFF1 did not bind to mucins purified from healthy or inflamed human colonic tissue (Figure 2). The specificity of binding of TFF1 to human gastric mucins and the lack of association with mucins isolated from other locations in the human GIT, parallels the expression pattern of TFF1 in the human GIT in vivo [14]. 

### 3.2. The Interaction of H. pylori with Human Gastric Mucins Correlates Closely with TFF1 Binding

**Figure 3:**
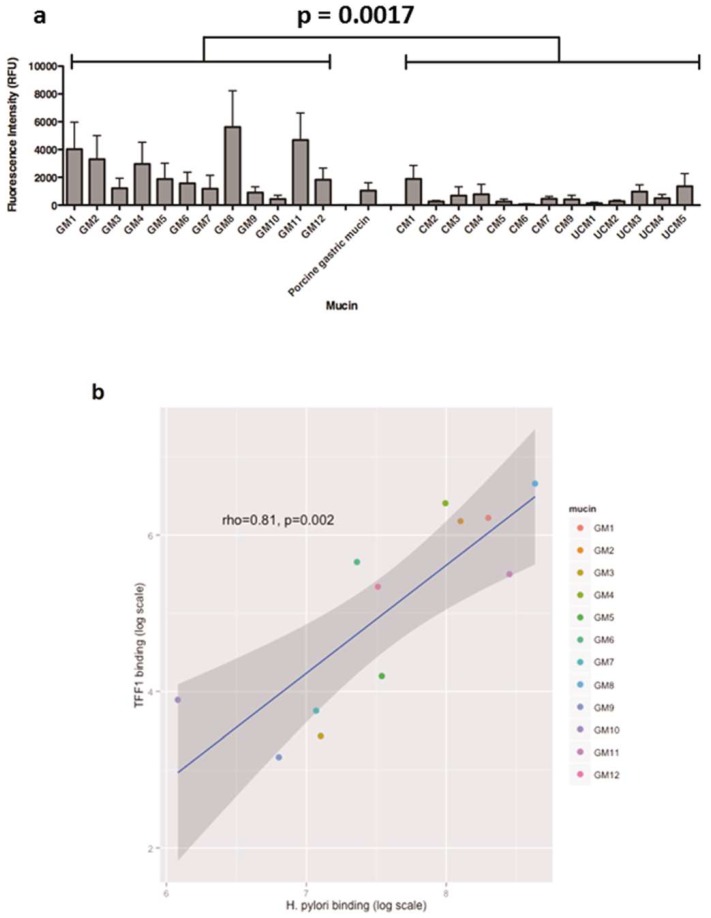
Correlation between *H. pylori* strain P12 and TFF1 binding of human gastric and colonic mucins. (**a**) Binding of *H. pylori* strain P12 to human gastric and colonic mucins. The average values from three biological replicates are shown, with the level of binding indicated by the fluorescent intensity and error bars representing mean ± standard deviation. The level of *H. pylori* binding to gastric mucins was significantly more intense than *H. pylori* binding of colonic mucins (*p* = 0.0017, Mann-Whitney U test). (**b**) *H. pylori* binding of human gastric mucins was plotted against TFF1 binding and the correlation between the two was determined by calculating Spearman’s correlation co-efficient.

To assess if there was a correlation between binding of *H. pylori* and binding of TFF1 to human gastric mucins, the binding of fluorescently-labelled *H. pylori* strain P12 to the same panel of gastric mucins was determined. Similarly to TFF1 binding, binding of *H. pylori* to the mucins varied depending on the individual gastric mucin sample. Some binding to colonic mucins was detected (Figure 3a), however *H. pylori* binding to gastric mucins was significantly more intense than that of *H. pylori* binding to colonic mucins (*p* = 0.0017, Mann-Whitney U test). No difference between binding to mucins isolated from healthy and inflamed colonic tissue was observed (*p* = 0.75 Mann-Whitney U test). 

Both TFF1 and *H. pylori* bound intensely to gastric mucins, in particular GM1, GM2, GM4, GM8, GM11 and GM12 with greatest binding intensity observed with GM8. The adhesion of *H. pylori* to gastric and colonic mucins was compared to the TFF1 interaction with the ggplot2 package in R. There was a strong correlation between the gastric mucins to which TFF1 bound and those to which *H. pylori* strain P12 bound (rho = 0.81), *p* = 0.0007; Figure 3b and Table 1). The congruent interaction suggests that TFF1 and *H. pylori* might bind to similar structures of gastric mucins. Due to the lack of binding of TFF1 to colonic mucins, there was no association between TFF1 and *H. pylori* binding to colonic mucins (rho = 0.37, *p* = 0.2). 

### 3.3. The Interaction of Various H. pylori and C. jejuni Strains to Human Gastric Mucins and TFF1

**Figure 4:**
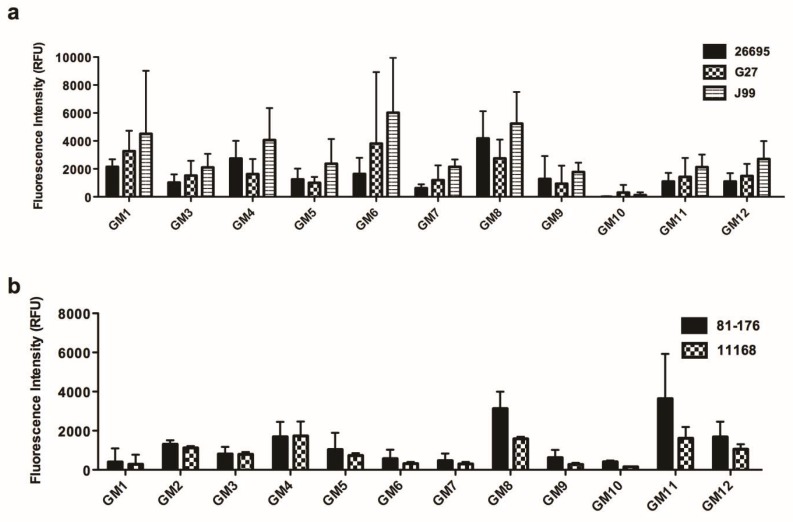
Binding of *H. pylori* and *C. jejuni* to human gastric mucins. Mucin microarrays containing human gastric (GM) mucins were probed with (**a**) *H. pylori* strains 26695, G27 and J99 and (**b**) *C. jejuni* strains 81-176 and 11168. The level of binding is indicated by the fluorescence intensity. The average values from three biological replicates ± standard deviation are shown. Gastric mucin 2 (GM2) was not printed on the arrays used in (**a**).

The binding of three other *H. pylori* strains and for comparison purposes two *C. jejuni* strains to gastric mucins was assessed to investigate if their binding also correlated with that of TFF1. Strains J99 and G27 both express functional adhesins which bind to the Le^b^ and H blood group antigens and to sialyl Le^x^ whereas strain 26695 does not bind to these structures [29] due to lack of functional BabA and SabA adhesin proteins [35]. All strains tested in this study (P12, 26695, J99 and G27) have been previously shown to bind to LacdiNAc [9]. Strain J99 bound with the highest intensity to all gastric mucins tested (Figure 4a). Strains J99 and G27 bound to mucins from secretor individuals (Le^b^ positive) with greater intensity than strain 26695, which corresponds to Le^b^ being the preferred ligand for the BabA adhesin although this result was not statistically significant (*p* ≥ 0.05, Mann-Whitney U test). For mucins from Le^b^ negative individuals the binding of strain 26695 was comparable with that of strain G27. Of note, all strains bound to gastric mucins with the same relative intensities which indicates that adherence to mucin is probably not dependent on a single adhesin for any one strain. As with strain P12, there was a strong correlation between binding of strains 26695, J99, G27 and TFF1 binding to gastric mucins (Table 1). No correlation was noted when binding of either of two strains of *C. jejuni* to gastric mucins (Figure 4b) was compared with binding of TFF1 (Table 1).

### 3.4. Binding of TFF1 and H. pylori to Gastric Mucins Correlates with Binding of the Lectin GS-II

**Figure 5:**
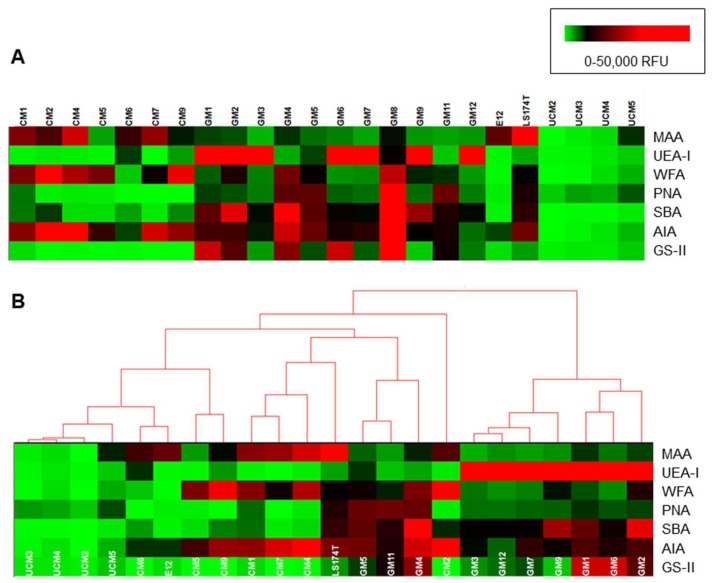
Lectin glycosylation profiles of human gastrointestinal tract (GIT) mucins. (**A**) Heat maps of medians of individual technical replicates plotted and scaled to a maximum intensity of 50,000 relative fluorescence units (RFU). (**B**) Heat map with dendrograms of hierarchical clustering generated using HCE 3.0. Color indicates fluorescence intensities as in the legend, with low intensity depicted as green, medium intensity in black and high intensity in red. MAA = *Maackia amurensis* agglutinin, UEA-I = *Ulex europaeus* agglutinin I, WFA = *Wisteria floribunda* agglutinin, PNA = Peanut agglutinin, SBA = Soya bean agglutinin, AIA = *Artocarpus integrifolia* agglutinin, GS-II = *Griffonia simplicifolia* lectin-II.

Interrogation of the human mucin microarrays with lectins demonstrated that the glycosylation of gastric mucins differed from that of the colonic mucins (Figure 5). Fucosylation, as demonstrated by UEA-I binding, was mainly present in the gastric mucins. Non-sialylated *O*-linked oligosaccharides, indicated by peanut agglutinin (PNA) binding, were detected in gastric mucins GM4, GM5, GM8 and GM11. Sialylation indicated by *Maackia amurensis* lectin agglutinin (MAA) binding and sulfation as indicated by *Wisteria floribunda* agglutinin (WFA) was most prevalent in the colonic mucins. Colonic mucins also had low or no soya bean agglutinin (SBA) binding, which indicated a lower overall quantity of accessible *N*-acetylgalactosamine (GalNAc) in colonic mucins compared to gastric mucins. There was a correlation between binding of MAA, which recognizes carbohydrate structures that contain α-(2,3)-linked sialic acid, and binding of TFF1 and *H. pylori* strains P12 and 26695 (Table 2). Notably there was a strong correlation between binding of TFF1 and binding of each of the *H. pylori* strains with binding of *Griffonia simplicifolia* lectin-II (GS-II) (Table 2) which is specific for terminal non-reducing α- or ß-linked *N*-acetylglucosamine (GlcNAc).

## 4. Discussion

TFF1 is co-expressed with the gastric mucin MUC5AC by mucus secretory cells of the gastric mucosa [14]. Previous work has shown that TFF1 is packaged within the secretory vesicles of gastric mucus granules with MUC5AC and that they are released together from the vesicles into the adherent mucus layer [19]. TFF1 is intimately associated with gastric mucus in vivo [15] and gel filtration under physiological conditions, and co-immunoprecipitation studies revealed a direct interaction between endogenous TFF1 and the gastric mucin MUC5AC, which was partially dependent on ionic interactions and the presence of divalent cations [19]. In the present study, the mucins present on the microarray were extracted from mucus by treatment with the chaotropic salt GuHCl. Mucin is insoluble in GuHCl while other proteins are soluble. Incubation with TFF1 dimer in vitro demonstrated that the dimeric form of TFF1 added exogenously interacted with gastric mucin that had been treated with GuHCl, which indicated the continued availability of the mucin ligand(s) for TFF1. Our results demonstrated that the interaction of TFF1 with mucin in humans is likely to be specific for gastric mucin as no interaction with any of the colonic mucins purified from 14 individual donors was detected. 

The specific interaction of TFF1 with gastric mucins reflects the in vivo localization of TFF1 in the GIT [14,36] and this direct binding to gastric mucins could be a way of localizing TFF1 within gastric mucus layer. Trefoil factors are localized within mucin granules in mucus-secreting epithelial cells and are secreted with mucins out of the cell and into the mucus layer [15]. The close association of trefoil factors with specific mucins in the GIT and evidence that TFF1 interacts with gastric mucin (this study and [19]) supports the hypothesis that TFF1 plays a central role in maintaining mucus integrity. 

Two of the three animal mucins that TFF1 bound most intensely to were gastric mucins. Interestingly, we have shown previously that *H. pylori* also adheres to the rat and bovine mucins to which TFF1 binds [29], although natural *H. pylori* infection does not occur in either rats or cattle. Both murine and porcine experimental models of *H. pylori* infection exist, but TFF1 did not bind at all to murine mucin, and binding to porcine mucin was modest. The physiological conditions in the stomachs of different animals may explain the lack of infection with *H. pylori* in species other than primates. Interestingly, TFF1 did not bind to mucin from the either of the colonic cell lines E12 or LS174T. This is in keeping with lack of binding of TFF1 with colonic mucins. However we have shown previously that E12 cells secrete TFF1, and it can be detected in the adherent mucus layer formed by the cells [20]. In addition, upon infection of E12 cells *H. pylori* can co-localize with TFF1 present in the adherent mucus layer and mutants unable to bind TFF1 show reduced infection of these cells [20]. These results suggest that *H. pylori* may bind to either free TFF1 or to TFF1 that is bound to gastric mucin. 

As TFF1 exhibited a similar binding profile to gastric mucins as *H. pylori* and both correlated well with the binding of GS-II, the binding of both TFF1 and *H. pylori* to gastric mucins may both be glycosylation dependent. GS-II binds to terminal α- or β-linked GlcNAc and this terminal residue may be the ligand recognized by both TFF1 and *H. pylori*. Indeed, the binding of TFF1 to ovomucoid at high concentration may support this hypothesis, as ovomucoid displays many GlcNAc terminated structures [37]. Our previous finding that TFF1 interacted with the core oligosaccharide of rough form LPS of *H. pylori* and that the interaction can be inhibited with free mannose and glucose demonstrated that TFF1 can indeed bind to carbohydrates [13]. The variability in binding of TFF1 to gastric mucins from different individuals suggested that binding is probably mediated via glycans present on the mucin; glycosylation is known to vary between individuals and lectin profiling of the gastric mucins analyzed in this study demonstrated differences in mucin glycosylation from different individuals. Previously, yeast two hybrid analysis has suggested that TFF1 binds to von Willebrand factor domains of mucins [38]. The results of this study do not rule out binding of TFF1 to von Willebrand factor domains but as these domains are highly conserved amongst secreted mucins [39] binding solely to Von Willebrand factor domains would not explain the variability in binding between different mucins unless variation in glycosylation resulted in exposure of the domains in some mucins. 

Interestingly an α-(1-4)-linked GlcNAc terminated *O*-linked oligosaccharide purified from gastric mucin has recently been shown to inhibit binding of *H. pylori* strains, including strain J99, to gastric tissue [40]. Oligosaccharides with terminal α-(1-4)-linked GlcNAc residues are found on the gastric mucin MUC6 expressed down in the glands [41] and they have antimicrobial activity against *H. pylori*, inhibiting biosynthesis of cholesteryl-α-D-glucopyranoside, a major cell wall component [42,43]. Only a small percentage of *H. pylori* infections result in serious disease such as duodenal ulceration (10–15%) or gastric cancer (1%) with the majority of infections being asymptomatic. Infection usually occurs early in life (<2 years of age) [2] and can reside in the human host for decades. Why *H. pylori* is so successful at causing persistent infection is not known. Recently, it has been shown that in asymptomatic *H. pylori* infections the expression of terminal non-reducing α-linked GlcNAc residues on the oligosaccharides of surface mucin is increased [40]. Interestingly TFF2, with which *H. pylori* does not interact and which is co-expressed in the stomach with MUC6, has been shown to have lectin activity and to bind to α-(1,4)-linked GlcNAc terminated oligosaccharides [44]. TFF1 binding to terminal α-linked GlcNAc residues was not demonstrated explicitly in this study. In addition, the potential role of β-linked GlcNAc, to which GS-II also binds, in mediating TFF1 and *H. pylori* binding to gastric mucin cannot be overlooked. Of note, there was also some interaction of GS-II with colonic mucins that were not bound by TFF1, but these interactions were not as intense as those observed with the gastric mucins (Figure 5). 

There was a correlation between TFF1 binding to gastric mucin and binding of the lectin MAA which has a specificity for α-(2,3)-linked sialic acid or Gal-3-SO_4_^2−^. *H. pylori* strains that express SabA can bind to sialyl Le^a^ and sialyl Le^x^, both found in inflamed tissue, [10] and expression of sialylated structures induced upon inflammation promotes chronic infection by increasing the number of ligands for *H. pylori*. In patients with intestinal metaplasia, a precursor to gastric cancer, an increase in blood group and Lewis antigen structures as well as LacdiNAc glycans and sialylated structures was found, all of which can act as ligands for *H. pylori* [40]. The results here suggest that further studies and more detailed and precise analysis of the glycans that mediate binding of TFF1 to gastric mucins is warranted. 

**Figure 6:**
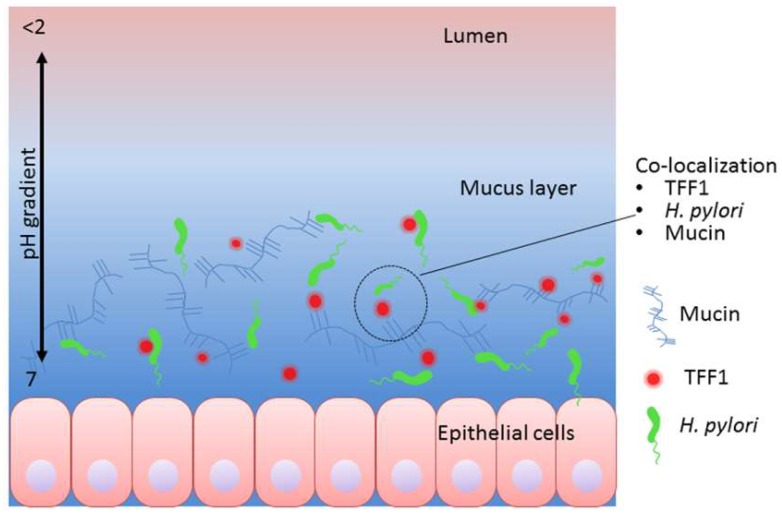
Suggested mechanism whereby TFF1 may help locate *H. pylori* in gastric mucus. Both TFF1 and *H. pylori* can interact with gastric mucin and with each other. Given that TFF1 has been shown to have two putative receptor ligand domains [18], that it interacts with gastric mucin [19] and that optimal interaction with *H. pylori* occurs at pH 5–6 [13], we suggest that TFF1 may locate *H. pylori* in gastric mucus close to the epithelial surface. The diagram shows *H. pylori* interacting with free TFF1 both, with TFF1 bound to gastric mucin and directly to gastric mucin. Interaction of *H. pylori* with TFF1 in gastric mucus may reduce the number of organisms that bind to epithelial cells thus minimizing development of inflammation and disease.

In summary, we show that TFF1 binds to gastric mucin and that there is a striking correlation between the mucins that bind TFF1 and those that bind *H. pylori*. The strong correlation between binding of GS-II to gastric mucin and binding of TFF1 and *H. pylori* suggest that both TFF1 and *H. pylori* may bind structures that contain α- or β-linked GlcNAc or that are co-expressed with α- or β-linked GlcNAc. Binding of TFF1 to gastric mucins was more exclusive than the binding of *H. pylori* and this finding, together with the strict co-localization of TFF1 with the gastric mucin MUC5AC in the stomach, suggests that the interaction of *H. pylori* with TFF1 may contribute to the distinct tropism that the organism displays for gastric mucin in vivo. Of interest in the murine experimental model of *H. pylori* infection, the presence of TFF1 is not necessary for infection, as *H. pylori* has been shown to infect *tff1*-deficient mice [45,46]. However, the absence of TFF1 resulted in severe inflammation and increased incidence of gastric tumorigenesis compared to infection with wild type mice [45,46]. This finding indicates that TFF1 may play an important protective role in preventing inflammation and the development of gastric cancer in *H. pylori* infections. Future in vivo studies should investigate if the presence of TFF1 and its interaction with *H. pylori* serves to locate the majority of *H. pylori* in the gastric mucus layer rather than deeper down at the epithelial cell surface. Such a localization of *H. pylori* would limit its direct interaction with the epithelial cells and the subsequent host pathogen signaling events that promote inflammation (Figure 6). 

## Figures and Tables

**Table 1:** Correlation between *H. pylori* strains P12, 26695, G27 and J99 and *C. jejuni* strains 11168 and 81-176 and TFF1 binding of human gastric mucins. *H. pylori* and *C. jejuni* binding of human gastric mucins was plotted against TFF1 binding and the correlation between the two was determined by calculating Spearman’s correlation co-efficient (rho) and *p* value.

Bacterial Strain	Rho Value	*p* Value
*H. pylori* P12	0.82	0.002
*H. pylori* 26695	0.74	0.01
*H. pylori* G27	0.72	0.02
*H. pylori* J99	0.82	0.004
*C. jejuni* 11168	0.39	0.21
*C. jejuni* 81-176	0.58	0.052

**Table 2:** Correlation between lectin binding and TFF1 and *H. pylori* binding to human gastric mucins.

Lectin	TFF1		*H. pylori* P12		*H. pylori* J99		*H. pylori* 26695		*H. pylori* G27	
	rho	*p*	rho	*p*	rho	*p*	rho	*p*	rho	*p*
MAA	0.636	0.03	0.734	0.009	0.436	0.18	0.636	0.04	0.591	0.06
PNA	0.566	0.06	0.769	0.005	0.464	0.15	0.491	0.13	0.473	0.14
WFA	0.308	0.33	0.482	0.11	0.209	0.54	0.536	0.09	0.273	0.42
SBA	0.392	0.21	0.587	0.05	0.300	0.37	0.636	0.04	0.327	0.33
UEA-1	−0.252	0.43	−0.189	0.55	0.364	0.27	−0.064	0.86	0.191	0.57
GS-II	0.811	0.002	0.769	0.005	0.791	0.006	0.718	0.01	0.800	0.005
AIA	0.454	0.14	0.559	0.06	0.454	0.16	0.500	0.12	0.482	0.14

Binding of TFF1 and all *H. pylori* strains to gastric mucins correlated with binding of lectin GS-II (indicated in bold). Spearman’s correlation coefficient (rho) and the *p* value are given.

## Supplementary Materials

The following are available online at http://www.mdpi.com/2076-2607/6/2/44/s1, Table S1: Blood group status of individuals from whom gastric mucus was obtained for mucin purification; Table S2: Print list for the animal gastrointestinal mucin microarrays; Table S3: Print list for the human colonic and gastric mucin microarrays; Table S4: Lectins used to characterize the glycosylation of gastric and colonic mucins, their binding specificity and the concentrations used (mg/mL).
