# Supplementary material for: Binding of Helicobacter pylori to Human Gastric Mucins Correlates with Binding of TFF1
Source: Microorganisms. 2018 May 18;6(2):44. doi: 10.3390/microorganisms6020044 (PMC6027488; doi:10.3390/microorganisms6020044)
Supplement: Supplementary file 1 [file microorganisms-06-00044-s001.pdf]

Supplementary Information

Binding of *Helicobacter pylori* to Human Gastric Mucins Correlates with Binding of TFF1

Table of contents

Table S1. Blood group status of individuals from whom gastric mucus was obtained for mucin purification S-2

Table S2. Print list for the animal gastrointestinal mucin microarrays S-3

Table S3. Print list for the human colonic and gastric mucin microarrays S-4

Table S4. Lectins used to characterise the glycosylation of gastric and colonic mucins, their binding specificity and the concentrations used (mg/mL). S-5

Supplementary Table S1. Blood group status of individuals from whom gastric mucus was obtained for mucin purification.

Gastric mucin	Blood group of donor
GM1	OLe^{b}
GM2	ALe^{b}
GM3	OLe^{b}
GM4	BLe^{a}
GM5	OLe^{a}
GM6	OLe^{b}
GM7	ND
GM8	OLe^{a-}
GM9	ALe^{a}
GM10	ALe^{b}
GM11	OLe^{a-}
GM12	OLe^{d} (a-,b-)

ND = not determined

Supplementary Table S2. Print list for the animal gastrointestinal mucin microarrays. Purified mucins were printed at various concentrations (mg/mL) in PBS supplemented with various concentrations of Tween 20 (%T). Different colours indicate same species.

Row	Number	Source	Concentration	Print buffer
1	6	Equine Stomach 6	0.25	PBS 0.01% T
2	10	Ovine Abomasum Antrum 10	0.1	PBS 0.01% T
3	11	E12	0.5	PBS 0.025% T
4	12	Ovine Descending Colon 12	0.15	PBS 0.01% T
5	13	Ovine Ileum 13	0.15	PBS 0.01% T
6	18	Ovine Spiral Colon 18	0.5	PBS
7	71	Chicken Proximal Small Intestine 34	0.25	PBS
8	35	Ovine Jejunum 35	0.5	PBS 0.01% T
9	36	Ovine Duodenum 36	0.15	PBS 0.01% T
10	37	Porcine Gastric Mucin 37	0.33	PBS 0.01% T
11	41	Chicken Large Intestine 41	0.2	PBS 0.01% T
12	52	Equine Duodenum 52	0.3	PBS 0.01% T
13	55	Deer Jejunum 55	0.25	PBS 0.025% T
14	56	Deer Large Intestine 56	0.75	PBS 0.025% T
15	57	Bovine Abomasum 57	0.25	PBS 0.01% T
16	58	Bovine Duodenum 58	0.5	PBS 0.01% T
17	59	Equine Small Intestine 59	0.25	PBS
18	60	Equine Left Ventral Colon 60	0.25	PBS 0.01% T
19	61	Bovine Spiral Colon 61	0.25	PBS 0.01% T
20	62	Deer Duodenum 62	0.5	PBS 0.025% T
21	65	Equine Right Ventral Colon 65	0.15	PBS 0.01% T
22	66	Equine Dorsal Colon 66	0.25	PBS 0.01% T
23	67	Deer Abomasum 67	0.25	PBS 0.01% T
24	70	Chicken Cecum 70	0.5	PBS 0.01% T
25	204	LS174T 72	0.5	PBS 0.01% T
26	85	Porcine descending colon	0.5	PBS 0.025% T
27	85	Porcine descending colon	0.6	PBS 0.025% T
28	86	Porcine jejunum	0.25	PBS
29	87	Porcine spiral colon	0.5	PBS 0.025% T
30	87	Porcine spiral colon	0.6	PBS 0.025% T
31	102	Porcine stomach	0.5	PBS 0.025% T
32	103	Porcine ceca	0.5	PBS 0.025% T
33	105	Mouse large intestine	0.4	PBS 0.025% T
34	106	Mouse cecum	0.3	PBS 0.025% T
35	107	Mouse stomach	0.5	PBS 0.025% T
36	121	Mouse small intestine	0.25	PBS 0.025% T
37	139	Rat ileum	0.5	PBS 0.025% T
38	140	Rat duodenum	0.5	PBS 0.025% T
39	141	Rat cecum	0.5	PBS 0.025% T
40	146	Rat stomach	0.5	PBS 0.025% T
41	147	Rat colon	0.75	PBS 0.01% T
42	ASF	Asialofetuin	0.5	PBS
43	RB	RNase B	0.5	PBS
44	Fetuin	Fetuin	0.5	PBS
45	Xferrin	Transferrin	0.5	PBS
46	Ovomuc	Ovomucoid	0.5	PBS
47	PBS	PBS		
48	PBST	PBS 0.025% Tween 20		

Supplementary Table S3. Print list for the human colonic and gastric mucin microarrays. Purified mucins were printed at various concentrations (mg/mL) in PBS supplemented with 0.01 to 0.025% Tween 20 (%T).

Row	Code	Sample	Print buffer	Conc
1	Muc1_0.5	Muc1	PBS 0.025% T	0.5
2	Muc2_0.5	Muc2	PBS 0.025% T	0.5
3	Muc3_0.5	Muc3	PBS 0.025% T	0.5
4	Muc4_0.5	Muc4	PBS 0.01%T	0.5
5	Muc5_0.5	Muc5	PBS 0.025% T	0.5
6	Muc6_0.5	Muc6	PBS 0.025% T	0.5
7	Muc7_0.5	Muc7	PBS 0.01%T	0.5
8	Muc9_0.5	Muc9	PBS 0.01%T	0.5
9	PBS_0.01%T		PBS 0.01%T	
10	GM1	Gastric mucin 2	PBS	0.5
11	GM2	Gastric mucin 4	PBS 0.025% T	0.5
12	GM3	Gastric mucin 11	PBS 0.01%T	0.5
13	GM4	Gastric mucin 4919	PBS 0.01%T	0.5
14	GM5	Gastric mucin 5945	PBS 0.01%T	0.5
15	GM6	Gastric mucin 5975	PBS 0.01%T	0.5
16	GM7	Gastric mucin 5878	PBS 0.01%T	0.5
17	GM8	Gastric mucin 6077(2)	PBS	0.5
18	GM9	Gastric mucin 6335	PBS 0.01%T	0.5
19	GM10	Gastric mucin 6894	PBS 0.025% T	0.5
20	GM11	Gastric mucin 6077	PBS 0.01%T	0.5
21	GM12	Gastric mucin 4893	PBS 0.01%T	0.5
22	PBS		PBS	
23	E12_M11	E12	PBS 0.025% T	0.5
24	LS174T_M72	LS174T 72 or 204	PBS 0.01% T	0.5
25	Porcine stomach mucin M102	Porcine stomach mucin	PBS 0.025% T	0.5
26	Chicken proximal small intestine M71	Chicken Proximal Small Intestine 34	PBS	0.25
27	Chicken large instestine mucin M41	Chicken Large Intestine 41	PBS 0.01% T	0.2
28	Chicken cecum_M70	Chicken Cecum 70	PBS 0.01% T	0.5
29	PBS_0.025%T		PBS 0.025% T	

Supplementary Table S4. Lectins used to characterise the glycosylation of gastric and colonic mucins, their binding specificity and the concentrations used (mg/mL).

Lectin	Abbreviation	Binding specificity	Concentration
Ulex europaeus agglutinin I	UEA-I	Fuc-α-(1→2), H type 2 antigen	10
Maackia amurensis agglutinin	MAA	Neu-α-(2→3)-GalNAc, Gal-3-SO_4^{2-} > Lac	10
Griffonia simplicifolia lectin-II	GS-II	Terminal non-reducing α- or β-linked GlcNAc	10
Peanut agglutinin	PNA	Gal (Gal-β-(1→3)-GalNAc (T-antigen) > GalNAc > Lac > Gal, terminal β-Gal)	15
Artocarpus integrifolia agglutinin	AIA	Gal, Gal-β-(1→3)-GalNAc (T-antigen), Gal-α-(1→6), sialylation independent.	15
Soya bean agglutinin	SBA	GalNAc > Gal	15
Wisteria floribunda agglutinin	WFA	GalNAc, GalNAc-α-(1→6)-Gal > GalNAc-α-(1→3)-GalNAc (Forsmann antigen) > GalNAc >> Lac > Gal, GlcA-α-(1→3)-GalNAc. Also binds chondroitin sulfate.	10
